# A State-of-the-Art Review on Structural Strengthening Techniques with FRPs: Effectiveness, Shortcomings, and Future Research Directions

**DOI:** 10.3390/ma17061408

**Published:** 2024-03-19

**Authors:** Muhammad Hammad, Alireza Bahrami, Sikandar Ali Khokhar, Rao Arsalan Khushnood

**Affiliations:** 1 NUST Institute of Civil Engineering, School of Civil and Environmental Engineering, National University of Sciences and Technology, Sector H-12, Islamabad 44000, Pakistan; 2 Department of Building Engineering, Energy Systems and Sustainability Science, Faculty of Engineering and Sustainable Development, University of Gävle, 801 76 Gävle, Sweden; 3 Bendcrete Construction Services (Pvt) Ltd., National Science and Technology Park, Sector H-12, Islamabad 44000, Pakistan

**Keywords:** FRP, near-surface mounted reinforcement, externally bonded reinforcement, section curvilinearization, external prestressing, CFRP, flexural strength, structural strengthening, epoxy, adhesive

## Abstract

In the pursuit of creating more sustainable and resilient structures, the exploration of construction materials and strengthening methodologies is imperative. Traditional methods of relying on steel for strengthening proved to be uneconomical and unsustainable, prompting the investigation of innovative composites. Fiber-reinforced polymers (FRPs), known for their lightweight and high-strength properties, gained prominence among structural engineers in the 1980s. This period saw the development of novel approaches, such as near-surface mounted and externally bonded reinforcement, for strengthening of concrete structures using FRPs. In recent decades, additional methods, including surface curvilinearization and external prestressing, have been discovered, demonstrating significant additional benefits. While these techniques have shown the enhanced performance, their full potential remains untapped. This article presents a comprehensive review of current approaches employed in the fortification of reinforced cement concrete structures using FRPs. It concludes by identifying key areas that warrant in-depth research to establish a sustainable methodology for structural strengthening, positioning FRPs as an effective replacement for conventional retrofitting materials. This review aims to contribute to the ongoing discourse on modern structural strengthening strategies, highlight the properties of FRPs, and propose avenues for future research in this dynamic field.

## 1. Introduction

**Figure 1:**
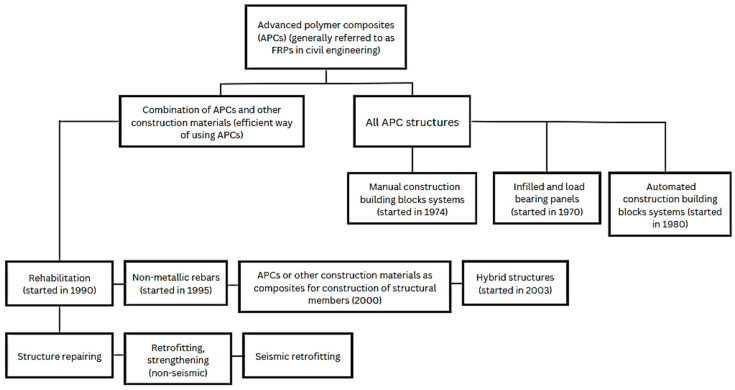
Utilization of FRPs in construction industry.

The evolution of the construction industry has witnessed a shift toward sustainable, eco-friendly, and cost-effective materials, replacing traditional alternatives [1,2,3,4,5]. While steel, a widely used and robust material, has historically served as a reinforcement in concrete, its limitations, primarily a lower strength-to-weight (s/w) ratio [5,6], prompted the exploration of alternative solutions. A significant advancement in the construction industry was the introduction of fiber-reinforced polymers (FRPs) as reinforcement bars by Russia in 1980 [7,8]. Figure 1 depicts the progress of fiber composite from the early 1970s to the 21st century [7].

One prevalent use of FRPs in civil engineering includes wrapping sheets of FRPs on the surfaces of reinforced cement concrete (RCC) members, steel, and masonry structures. This application aims to enhance the stiffness and strength of these structures, using FRP reinforcement bars within concrete members [9,10,11,12]. Notably, FRP-reinforced concrete (FRP-RC) structures exhibit eight times greater strength than conventional steel RC structures [6,13,14]. In Europe, studies on FRP reinforcements for the rehabilitation and strengthening of bridges started in the late 1980s [15]. The University of California, San Diego, conducted extensive testing to assess the effectiveness of FRPs in structural rehabilitation. The results indicated the efficacy of FRPs in strengthening of bridge piers [16,17]. Glass fiber-reinforced polymer (GFRP) has proven effective in strengthening of wood bridges, while carbon fiber-reinforced polymer (CFRP) overlays and sheets have successfully retrofitted prestressed concrete pipes affected by corrosion [16,18].

The exceptional performance of FRPs in structural rehabilitation encouraged further innovations, leading to the utilization of FRP shells and tubes in new composite structures [19,20]. Instead of relying on supplementary steel components for repair and strengthening, FRPs gained popularity among structural design consultants due to their outstanding characteristics including high strength, lightweight nature, and corrosion resistance [12,16,19,21,22]. Although FRPs gained popularity in Japan later than in other countries, it was the first country in the world to establish design guidelines for the use of FRPs in fortifying existing structures [23,24]. As the global usage of FRPs as a reinforcement material for structures increased, researchers worldwide revised and improved design philosophies [25].

Although FRPs are now becoming increasingly prevalent, there have been a few challenges in their use for repairing and strengthening of RCC structures owing to their variation in properties under high temperatures [26]. Therefore, multiple researchers carried out detailed investigations to study the response of FRPs under several temperature conditions [26,27,28,29,30,31]. The impressive results exhibited the ability of FRPs to improve the stiffness, ductility, and strength of structures. Researchers also explored the response of structures strengthened with FRPs under various loading conditions, including static and dynamic scenarios [32,33]. In recent years, RCC structures with embedded FRP reinforcements have gained popularity among civil engineers. Despite their advantages over conventional steel, codes and comprehensive guidelines are still not available for different types of FRP materials. Annex JA of Eurocode 2 [34] is a detailed annex that provides comprehensive guidelines for the design of new RCC structures with embedded FRP reinforcements. The embedded reinforcements can be bars, rods, or meshes. Being superior to others and most commonly used, only CFRP and GFRP are included in this annex. Additionally, in Annex R of Eurocode 2 [34], the design rules for the structural elements with embedded FRP reinforcements are discussed with minimum requirements, as elastic modulus should be $E_{fR}$ = 40,000 N/mm^2^, the ratio of $f_{tk.100a}$ (long-term tensile strength) to $E_{fR}$ should be greater than or equal to 0.005, the minimum bond strength for the long term ${(f}_{bd\cdot 100a})$ should be greater than or equal to 1.5 MPa, the longitudinal reinforcement ratio $\left(\rho_{lf}\right)$ for members should be less than or equal to 0.05, and the compressive strength (*f′_c_*) of RC should be equal to 20 MPa.

Researchers have proposed novel approaches, such as near-surface mounted (NSM) and surface curvilinearization (SC), for strengthening of existing structural elements as the use of FRPs for this purpose increased [35,36,37,38]. Azevedo et al. [39] studied the fire behavior of concrete slabs reinforced with CFRP sheets, taking into consideration three different approaches: NSM, EBR, and a novel technique called continuous reinforcement embedded at the ends (CREatE). The results displayed that CREatE without fire protection provided high fire resistance for about twenty-four minutes. However, with this protection, CREatE and NSM showed similar fire resistance for about 2 h. Ghaleh and Mostofinejad [40] compared EBR in grooves (EBRIG) and EBR on grooves (EBROG) strengthening methods using CFRP-RC specimens and performed single lap-shear tests. The outcomes revealed more noticeable improvements in bond properties by grooving methods (EBRIG and EBROG) than EBR. Additionally, EBRIG joints showed better performance compared to others. Also, the reason for the failure of EBROG joints was cohesive debonding, while for the EBRIG method, different failure modes were demonstrated, like the rupture of FRP sheets and deep debonding, etc. Additionally, Wysmulski [41] examined Z-shaped thin-walled carbon–epoxy laminated columns. Two different configurations were tested. The columns were loaded eccentrically in compression. For the finite element analysis, Abaqus software (https://www.3ds.com/products/simulia/abaqus) was used. The outcomes of his study enabled the evaluation of the effect of eccentric compressive loading on the buckling mode and critical force values.

Rozylo [42] compared the failure of two thin-walled composite CFRP columns with different cross-sections. Compressive loading was applied on specimens until the point of failure, and a few failure stages like crack initiation, delamination, and decrement in load-carrying capacities were observed. Moreover, finite element analysis considering various material damage models was conducted. The major failure was observed at the end section of the columns. On the outer flanges of the columns, the delamination phenomenon predominantly occurred.

**Figure 2:**
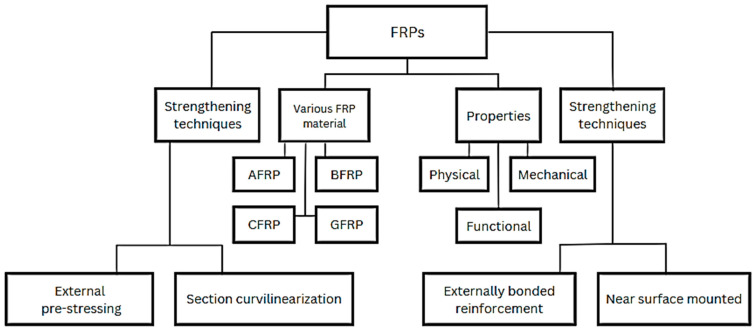
Layout of this review article.

The current article presents a comprehensive state-of-the-art review of these innovative strengthening techniques for both existing and new structural members utilizing FRP materials. Furthermore, it delves into the mechanical, physical, and functional properties of FRPs, including flexural and shear strengths, density, s/w ratio, temperature effects, and fire resistance. The review concludes by discussing the future research directions depicted in Figure 2 in detail.

## 2. Various FRP Materials

**Figure 3:**
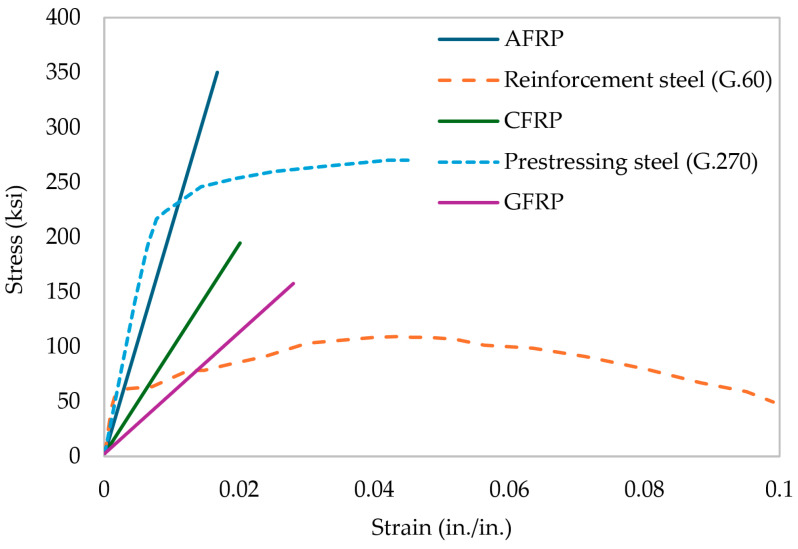
Comparison of steel with different FRP materials [50].

**Figure 4:**
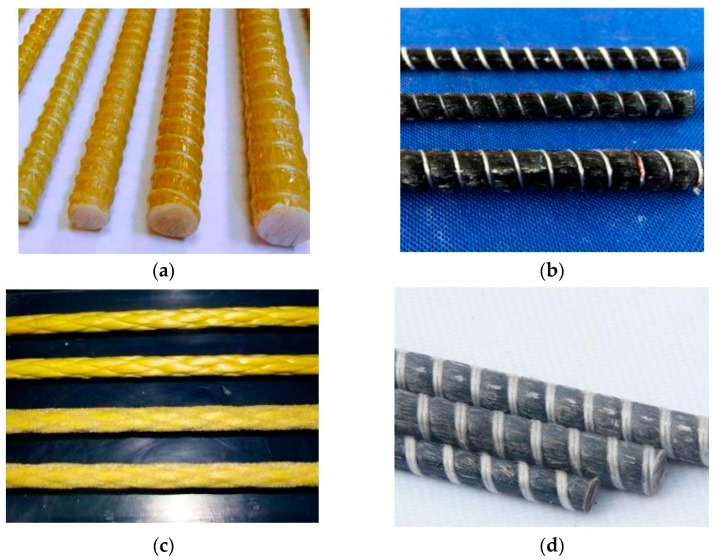
(**a**) GFRP bars [61], (**b**) CFRP bars [62], (**c**) AFRP bars [63], and (**d**) BFRP bars [64].

Conventional FRP reinforcements are made using the pultrusion method. Aramid fibers are used for aramid fiber-reinforced polymer (AFRP), basalt fibers for basalt fiber-reinforced polymer (BFRP), carbon fibers for CFRP, and glass fibers for GFRP [43]. Figure 3 illustrates a comparison between steel and different FRP materials. With high manufacturing capacity, E-GFRP hitherto was considered the most cost-effective composite compared to other FRPs typically utilized in the construction industry. AFRP is not a preferred type of reinforcement for civil applications due to its lower compressive strength [44,45]. Figure 4 illustrates four types of FRP rebars typically utilized in the construction industry: (a) GFRP, (b) CFRP, (c) AFRP, and (d) BFRP. The cost of BFRP is higher compared to GFRP due to the enhanced mechanical and durability properties such as high strength and alkali resistance, etc. [18]. CFRP is superior among all FRPs because of its high strength, resistance to creep failure, and better fatigue behavior. In addition, CFRP is widely available in a broader range of strengths and grades owing to different sources of carbon fibers and production processes [46,47,48,49].

GFRP consists of glass fibers to improve the strength and stiffness of plastics [51,52]. Table 1 lists the typical properties of GFRP. Composites consist of approximately 1–2% of glass fibers by weight [53]. The overall properties of GFRP bars are dependent upon several factors including reinforcing fibers, number of fibers, orientation of fibers, type of polymer matrix, bond between matrix and fibers, etc. [54]. Compared to conventional construction materials, GFRP has a high s/w value [12,55]. GFRP also exhibits properties like thermal insulation, thermal resistance, and resistance to alkaline conditions, chemicals, and salt water (brine) [56,57,58]. According to research works, GFRP creep strain is approximately 0.5–1%. GFRP is employed in the construction of bridges and structural members such as beams and columns, domes, and other non-structural elements. GFRP is cheaper than other FRP composites used in the construction industry [12,59,60].

CFRP is composed of carbon fibers which provide it high tensile strength. Additionally, CFRP has a very high modulus of elasticity and is very lightweight, approximately 60% lighter than steel with a similar strength [30,44,45,46,47,48,49,65,66,67]. Table 2 summarizes the typical characteristics of CFRP. This makes CFRP an ideal material for applications where high capacities and weight reduction are required. Carbon fibers present inside CFRP have diameters ranging between 4 and 10 μm. The fibers mainly consist of carbon atoms bonded in crystals, with alignment along the long axis, providing a high s/w value due to the crystal arrangement. According to ACI, the regular humidity and creep strain at 20 °C of FRP remains below 0.010% after 3000 h at load levels of 80% of the ultimate strength equivalent [68,69]. Although CFRP exhibits better strength and is utilized in structural strengthening and retrofitting works, its cost is approximately 5–10 times higher compared to other materials such as steel [48].

AFRP comprises heat-resistant synthetic aramid fibers. AFRP with aramid fibers demonstrates great strength and has properties such as a high elastic modulus, thermal resistance, and high resistance to alkaline environments, and has less density than glass fibers [56,70,71]. A few typical properties of AFRP are outlined in Table 3. AFRP has gained popularity among textile and fiber-reinforced composites. AFRP tends to absorb moisture and maintains sensitivity throughout the manufacturing process until it undergoes impregnation with a polymer matrix [72]. The breaking point of AFRP under a high load rate is approximately 35–40% higher than CFRP, GFRP, or BFRP, resulting in only a 13% strength reduction after 100,000 loading cycles [73]. Based on the research, AFRP creep strain is approximately 0.5–1% [74]. Although AFRP has high tensile strength, its use is limited in concrete structures with high compressive loadings owing to its low compressive strength [75]. Finally, comparing the cost of AFRP with the rest of FRPs, AFRP is less expensive than CFRP but costlier than GFRP.

BFRP consists of micro basalt fibers with diameters ranging between 15 and 20 μm. These fibers are made up of minerals like olivine, pyroxene, and plagioclase. Basalt is a natural mineral present in significant amounts inside the crust of the earth. BFRP shows properties such as alkali resistance, high strength, improved thermal conductivity, and high resistance to corrosion, heat, and chemical effects, high tensile strength, great elongation at fracture, etc. [31,76]. Some of the most significant properties of BFRP are summarized in Table 4. The estimated strength retention of the bars, which have been in service for 50 years, varies between 70% and 90% in different environments such as moist or dry conditions, with average yearly temperatures ranging from 5 °C to 35 °C. The high load-bearing capacity makes BFRP very useful for automobiles (vehicles) and construction industries (structural strengthening) [31].

## 3. Physical Properties

FRPs are gaining popularity in the construction industry because of their extraordinary physical properties like the *s*/*w* ratio, rigidity, density, and stiffness [6,73,77,78]. Structural engineers started using them as a replacement for conventional steel reinforcements. This review article is about the recent advancement of FRPs; therefore, a few of the properties are explained here. A few physical properties of FRP composites are given in Table 5.

### 3.1. Strength-to-Weight Ratio

FRPs have become very valuable assets to the construction industry, thanks to their amazing physical properties [79]. FRPs have been employed for structural repair and strengthening instead of conventional reinforcements due to their high s/w ratio [23,73,80]. The high strength of FRP with the remarkable mass reduction makes them useful for structural applications. FRPs (CFRP and GFRP) have a 3–7 times higher s/w ratio and 70–80% less weight compared to that of steel [7,81]. With high flexibility, FRPs have been used for structural fortification in different straight, curved, and irregular places [82]. The fiber content in the composite is a very important factor in determining the characteristics of the composite. Increasing the amount of fiber to the optimum value results in an enhanced mechanical property, while excess fibers can affect the properties of FRP in a negative way [83]. Glass fiber is considered a durable and lightweight material. Generally, FRP indicates high resistance to chemical attacks compared to conventional steel rebars [56,72,84,85,86,87,88]. However, chemical resistance tests, specifically through NaOH treatment, on mixed composites of carbon and sisal fibers with varying fiber–weight ratios revealed that the hybrid composite is not resistant to carbon tetrachloride [84]. In accordance with studies, aramid fibers have a lower s/w ratio than other FRPs. GFRP and CFRP have similar s/w ratios [70,73]. When analyzed in the direction of the fiber alignment, both carbon and aramid fibers display a higher s/w ratio. In comparison, glass has a relatively lower s/w ratio, though it remains fairly high, akin to carbon or aramid [7,89]. Conversely, basalt fibers present a superior s/w ratio and Young’s modulus compared to E-glass fibers. A high s/w ratio is a crucial characteristic of FRP, making it useful for civil engineering applications.

### 3.2. Density

FRPs with low density and high strength were utilized for space research in the 1960s–1970s. However, as the density of all used composites is lower than that of steel, the material’s bulk density is not a significant concern. [90]. Despite the advantages of natural fibers over fabricated materials, such as their low density and cost-effectiveness, they are constrained by a limitation in processing temperature [91,92]. The strongest composites are those that have undergone curing and have a high density and Young’s modulus. However, heat treatment removes the binders, leaving a less dense and more brittle composite [93]. Basalt plastic sheets have a density ranging between 1350–1380 kg/m^3^ and a thickness of nearly 1.5–2.5 mm [94]. On the other hand, CFRP has a density of 1600 kg/m^3^. The basalt–epoxy composite can effectively reinforce the concrete members, exhibiting even more tensile strengths. The adhesive, known as Sikadure-30, has a high adhesive paste with moderate viscosity and a density of 1310 g/L. This resin blend is commonly associated with FRPs utilized for the fortification of concrete structures [95]. The density of any composite can be calculated by means of Equation (1) [89]:

(1) $$\rho_{c}\;=\;V_{f}\;\rho_{f}+V_{m}\;\rho_{m}$$

(2) $$V_{f}\;=\;\frac{\mathrm{relative}\;\mathrm{volume}\;\mathrm{of}\;\mathrm{fibers}}{\mathrm{Total}\;\mathrm{volume}\;\mathrm{of}\;\mathrm{laminate}}$$

(3) $$V_{m}\;=\;\frac{\mathrm{relative}\;\mathrm{volume}\;\mathrm{of}\;\mathrm{matrix}}{\mathrm{Total}\;\mathrm{volume}\;\mathrm{of}\;\mathrm{laminate}}$$

(4) $${\;V}_{c}\;=\;\frac{\rho_{c}\;-\;\rho_{exp}}{\rho_{c}}$$

where $\rho_{m}$ is the matrix density, $V_{f}$ is the volume proportion of fibers, $V_{c}$ is the void volume proportion, $V_{m}$ is the matrix volume proportion, and $\rho_{f}$ is the density of the laminate.

## 4. Mechanical Properties

Mechanical properties such as tensile, compressive, shear, and flexural strengths, elastic modulus, and creep rupture are important factors in distinguishing various FRP materials. However, in this article, some of the mechanical properties are discussed in detail. The mechanical properties of different FRPs are tabulated in Table 6.

### 4.1. Shear Strength

Enhancement of the shear strength of RC structures using externally applied FRP sheets is greatly influenced by the bonding performance at the point of contact between FRP sheets, thickness of FRP sheets, properties of concrete, total layers, and typology of epoxy materials with fibers [10,96,97]. As epoxy absorbs moisture, the shear strength of the strengthened concrete members tends to decrease [98,99]. However, the shear reinforcement (ties or stirrups) is important for structures reinforced with FRPs. In the design of RC, shear reinforcements result in deep cross-sections, particularly where shear is a critical factor [100]. While initially, a deeper member might not be the preferred choice, it may increase the element’s cracking moment. Having a cracking moment 25% more than the applied moment enables the utilization of the gross moment of inertia for deflection calculations and allows the entire concrete member section to withstand shear loadings [101]. Young’s modulus of most FRP bars is relatively low compared to that of steel rebars. A low Young’s modulus means low stiffness which leads to deep members [100]. Therefore, extra reinforcement is required to limit the crack widths and control the long-term deflections. Mistretta et al. [102] compared the shear capacity formulations provided by ACI, AFGC, and CNR to experimental outcomes on GFRP-RC beams. Their study concluded that all standards underestimated the shear capacity of beams, particularly for beams with less or minimum shear reinforcement. This experimental study illustrated that further research is required to assess the shear resistance and the effect of considering several parameters like strut inclination for better predictions. The optimization of structures may benefit from various methodologies based on the shear reinforcement percentage. Additionally, finite element models accurately copied the experimental behavior and offered the potential for the optimization of a material. To properly account for the GFRPs’ stirrup resistance and improve the design capacity, their study recommended considering different reduction coefficients. Following ACI 318 [103], shear strength design of FRPs can be performed. Meanwhile, it also facilitates the shear resistance of steel bars. However, ACI 440.1R-2015 [68] can also be used, but it does not allow for FRP’s dowel actions for the shear resistance. Furthermore, the predictive model of the shear resistance for FRP-RC beams in ACI 440.1R-2015 [68] was based on research by Tureyen and Frosch. The ACI 440.1R-2015 model was dependent on the parameters of the beam width (*b*), effective depth (*d*), compressive strength of concrete (*f′_c_*), reinforcement ratio (ρ*_f_*), and elastic modulus (*E_f_*) of FRP bars. Notably, the shear span-to-depth ratio (*a/d*) was not considered in this model. Shear contribution from FRPs is determined based on failure modes, with the maximum allowable strain set at 0.004 for FRP rupture-related failures. Moreover, the highest observed 45° strain reading at the beam sides for the sheet is 0.40%, contrasting with its ultimate strain of 1.2% [68]. Eurocode has also included several rules in Annex J for the fortification of existing concrete structures with CFRP. Being the most advantageous and common type of FRP, only the rules for CFRP are currently included in the annex. In addition, the two most common strengthening techniques, NSM and EBR, are considered in Annex J. Eurocode also provided additional parameters like bending radius, number of FRP sheets or strips, and lapping of a fully wrapped FRP system for shear strengthening of structures [34]. Furthermore, D’Antino and Triantafillou [104] evaluated five different design guidelines including EN-1998-3 [105], ACI 440.2 R-08 [106], TR-55 [107], DAfStb [108], and CNR-DT 200-R1.2013 [109]. They included a newly proposed model from German guidelines, similar to the model proposed in Annex J of Eurocode. The study involved the datasets of 229 RC beams strengthened in shear which failed in shear. Although the models were accurate for the open or U-shaped configuration of FRP, every model underestimated the shear strength of FRP with a fully wrapped configuration. In one study, the application of the ultimate strengthening of a hollow concrete bridge column resulted in the restoration of its ductility and strength, and the ratio of the flexural shear strength [110]. Some studies indicate that the impact of fiber shawls is less pronounced in filled columns compared to hollow columns. This effect is also observable in hollow columns, with GFRP being less effective than CFRP [78,80,111,112,113,114,115,116,117,118,119,120]. El-Sayed [121] discovered that the shear strength of concrete beams, wrapped by CFRP sheets, increases in parallel with the ultimate stiffness and strength of the control beam and the reduction in the ductility of a RC beam. Li et al. [122] introduced a novel approach for shear reinforcement in RC beams utilizing CFRP mesh fabric (CFRP-MF) instead of conventional steel stirrups. Fifteen beams were tested with CFRP-MF or steel stirrup reinforcement. The variables were the shear *a/d* ratio and CFRP-MF reinforcement ratio. The results demonstrated similar shear behavior between CFRP-MF and steel stirrup-reinforced beams. Faramarzi et al. [123] conducted experiments on un-strengthened T-beams as well as DE CFRP-strengthened T-beams. The main parameter of the study was the *a/d* ratio. The beams with an *a/d* ratio of 1.9 failed in terms of shear compression, whereas the corresponding tested beams with a ratio of 3 failed in terms of diagonal tension. An increment of the *a/d* ratio from 1.9 to 3 illustrated a reduction in total shear force capacity by about 27% for un-strengthened beams and 23% for strengthened beams, respectively. A reduction in the *a/d* ratio from 3 to 1.9 led to a decline in the shear resistance gain attributable to DE CFRP bars, dropping from 37.2% to 29.6%. Additionally, the TR55 design model displayed a significant underestimation of the total shear force capacity for the tested beams. Murad [124] evaluated different orientations of FRP used for the fortification of beams through EBR. Through experimentation, it was found that the 63° inclined CFRP had a remarkable impact on increasing the shear strength and ultimate deflection of the specimen. Nominal strength can be calculated by employing Equation (5):

(5) $$V_{n}\;=\;V_{s}+V_{c}$$

(6) $$V_{c}\;=\;2\sqrt{{f^{\prime }}_{c}}\;\cdot \;bd$$

(7) $$V_{c}\;=\;\frac{A_{s}\;f_{y}\;d\;}{s}$$

where $A_{s}$ is the flexural reinforcement area, *d* is the distance from the farthest compression fiber to the center of flexural reinforcement, *b* is the beam’s width, *s* is the horizontal spacing of stirrups, $f_{y}$ is the yield stress of reinforcement, and ${f^{\prime }}_{c}$ is the compressive strength of concrete.

Furthermore, section 8.2 of Eurocode 2 provides provision for the shear strengthening of concrete elements. Shear strengthening can be done by means of the NSM and EBR techniques. CFRP wrapping can be conducted on structural elements in several configurations such as closed wrapping, L and U-shaped strips, etc.

The shear strength of a concrete section strengthened by CFRP can be calculated as:

(8) $$\tau_{Rd,CFRP}=\tau_{Rd}+\tau_{Rd,f}\leq 0.5vf_{cd}$$

where $\tau_{Rd}$ is the design shear strength provided by section 8.2 of Eurocode 2.

(9) $$\tau_{Rd,f}=\frac{A_{f}}{S_{f}}\frac{f_{fwd}}{b_{w}}\;\left(cot\theta +cota_{f}\right)sina_{f}$$

(10) $$\frac{A_{f}}{S_{f}}=\left\{\begin{array}{c} \frac{2t_{f}b_{f}}{s_{f}}\;\;for\;discrete\;CFRP\;strips\\ 2t_{f}sina_{f}\;for\;discrete\;CFRP\;strips \end{array}\right.$$

In the above equation, $a_{f}$ is the angle between the longitudinal axis of the member and CFRP, $f_{fwd}$ is the CFRP’s design shear strength, and θ can be taken as 45° for the determination of $\tau_{Rd}$ and $\tau_{Rd,f}$. Table 7 provides a summary of a few selected publications.

### 4.2. Flexural Strength

FRP-reinforced elements typically have an extra reinforcement, wherein the proportion of the FRP bar to concrete exceeds the balanced ratio. Accordingly, the failure mode is predominantly controlled by crushing of concrete in the member. However, when the FRP-to-concrete ratio is below the balanced ratio, the failure mode shifts to the FRP rupture [81]. The reduction factor for the flexural strength is restrained within the range of 0.5 to 0.6, based on the ratio of the proposed reinforcement to the neutral reinforcement. This limitation arises due to the insufficient ductility in failure modes associated with FRP reinforcement. Specifically, the strength reduction factor for the FRP rupture is set at 0.55. However, in cases where failure occurs through concrete devastation, the attenuation factor for the flexural strength increases to 0.6 [125]. The flexural strength of the section covered with CFRP exhibits a clear decrement with the increment in the detachment factor. The flexural strength of FRPs is calculated through the ACI 440 standard, identical to ACI 318, because FRP rebars do not display yielding behavior like steel bars [68,126]. For flexural strengthening through the EBR technique, Annex J of Eurocode 2 also suggested that the maximum center-to-center spacing between FRP strips should not exceed 0.2 times the distance between zero moment points, 0.4 times 400 mm and the cantilever length, and three times the slab thickness. The distance from the surface of the concrete element to the edge of the strip must not be less than the concrete cover. Additional detailed guidelines such as the arrangement and placement of FRP reinforcements are discussed in the Fib bulletin [127]. Following some of these rules would help avoid the premature detachment of FRPs from the strengthened element.

Several investigations have explored the factors influencing the flexural strength of these composites, including the fiber length, content of the bonding agent, and pre-activation of the fibers before fabrication [12,35,84,128,129,130,131,132,133]. Notably, longer fibers, such as those with a length of 10 mm, result in greater distances between binder interaction points, leading to a composite with comparatively weaker properties [134]. Mastali and Dalvand [135] suggested that an increase in the carbon fiber content considerably improves the flexural performance of beams. Furthermore, the study reported the finding that the flexural strength of reinforcements of plain concrete beams demonstrated an approximate increase of 31%, 50%, and 67% with reused carbon fibers. Basalt and PVA fibers, employed through the EBR method, serve to provide substantial support and crack resistance. This strategy enhances the overall toughness of the matrix, resulting in a nearly 27% improvement in the flexural strength at the yielding zone [136]. Carbon fiber shows less strain compared to glass fiber, and CFRP exhibits low ductility compared to GFRP. Interestingly, the blend of glass and polyethylene fibers illustrated no detrimental effects on the flexural strength of the specimens [131]. Park and Jang [137] introduced a hybrid layered composite scheme comprising carbon fibers and polyethylene fibers embedded in an epoxy matrix. The strength of this hybrid composite is notably reliant on the arrangement of the reinforcing fibers. Placing CFRP at the marginal sheet greatly enhances the flexural strength. Additionally, increasing the length of recycled CFRP fibers in self-compacting concrete yields a substantial improvement in the flexural strength, with a recorded increase of almost 6% compared to the strength observed with a constant fiber length [135]. CFRP has been found to improve the ductility and shear strengthening in columns and beams. Further, it contributes to an increase in the flexural, shear, and torsional strengths, as mentioned by different researchers [138,139]. The computation of the cross-breaking strength of flexural-strengthened members is carried out using Equation (11) [140]:

(11) $$\mathrm{cross-breaking}\;\mathrm{strength}\;=\frac{1.5\;WL}{BD^{2}}$$

where *W* is the load in kN, *B* is the width, *D* is the thickness, and *L* is the span between two end supports. A summary of a few selected publications is listed in Table 8.

In addition, the tensile strength of concrete poses a limitation to the bond strength of FRPs because debonding of FRPs typically starts from shear cracking just under the FRP sheet or from the position of internal reinforcements. Moreover, the bonding strength does not increase with the increment in the length of the FRP sheet because, after the effective bond length, the bond strength remains unaffected. Hence, anchorage systems are recommended to improve the utilization of the tensile capacity of FRPs [141].

After detailed research, a few guidelines for the design of an anchorage system for CFRP sheets to strengthen the structural elements are proposed as follows. To reduce the stress concentration, rounding the edges of anchor holes in the structural member is recommended. With the insertion of the anchor system to a depth of approximately 15 cm to ensure a 5 cm depth of the anchor in concrete, the total cross-sectional area of all anchors in every row should be more than twice the longitudinal sheet’s cross-sectional area. Further research proposed additional guidelines as follows. To evenly distribute the forces across CFRP and concrete, dividing the anchor into smaller anchors is recommended. For effectiveness, multiple rows should be considered, and spacing of the anchor should be around 3.8 cm. The type and material properties of CFRP should be verified because they can impact the anchor’s strength [142].

**Table 8:** Summary of relevant studies on strengthening by FRP in flexure.

Author	Ref.	Considered Parameters
Arduini et al.	[143]	Typology of composites *a/d* Impact of initial cracking Surface conditioning
Ritchie et al.	[144]	Typology of composites
		Anchorage at end of plate
Arduini et al.	[145]	Number of composite plates
		Anchors at end of plate
He et al.	[146]	Anchors at end of plate
David et al.	[147]	Typology of composites
		Thickness and/or number of plies
Gangarao and Vijay	[148]	Number of plies
		Impact of initial cracking
		Anchoring by covering with CFRP sheets
Spadea et al.	[149]	External anchorage for CFRP plates
Garden et al.	[8]	*a/d*
		Anchorage at end of plate
Ross et al.	[150]	Current reinforcement ratio
		Impact of ratio of composite area to steel
Davies et al.	[45]	CFRP placement
		Anchorage with help of vertical sheets
Mastali and Dalvand	[135]	Different volume fractions of fibers
		Recycled CFRPs
Park and Jang	[131]	Orientation effect of polyethylene fibers
		Typology of composites

## 5. Functional Properties

The type of fiber is a very critical parameter in determining the functional properties of the composites. For instance, carbon fibers behave differently under elevated temperatures compared to glass fibers. Some of the functional properties of FRPs include elevated temperature effect, fire resistance, and electrical conductivity.

### 5.1. Temperature Effect

Experiments investigating the effects of temperature on materials such as FRPs often span five years. However, the predicted lifespan of infrastructures, such as bridges, extends beyond 50 years, making it challenging to effectively anticipate the long-term performance of FRP structures [151]. Concerning temperature effects, the mechanical performance experiences a significant decrease as the temperature reaches the transition temperature of glass (Tg) of FRPs [95,152,153,154,155]. Specifically, the variance in the mechanical response of BFRP, AFRP, CFRP, and GFRP rebars is studied under low temperatures ranging from −200 °C to 200 °C. Meanwhile, high temperatures are evaluated within the ranges of 180 °C to 600 °C [27,95,152,153,154,155,156,157,158,159,160,161,162,163,164]. In one study, it was noted that at room temperature, beams reinforced with FRP predominantly displayed flexural cracking in concrete, followed by a slight detachment of FRP before reaching the FRP rupture. The initial FRP rupture occurred when the external load reached 99.5% of the maximum load [165]. Karbhari et al. [166] subjected concrete cylinders with CFRP sheets as a reinforcement to two hundred freeze and thaw cycles ranging from −20 °C to 23 °C. The outcome was an abrupt rupture between concrete and reinforcement. Another research exposed 50 cylinders to 100 and 200 freeze and thaw cycles at temperatures of −20 °C and 20 °C, respectively, at 70% relative humidity. Except for the bar with a diameter of about 20 mm, which indicated a 15% reduction after 200 cycles, the outcomes demonstrated almost no effect on the joint strength values. Zhao et al. [167] conducted a study on the fatigue behavior of FRP-reinforced beams at elevated temperatures and claimed that elevated temperature exposure remarkably impacted FRP-RC beams during the initial fatigue cycle, causing degradation in both concrete and FRP bars resulting in the reduced cracking load alongside increased crack width and deflection. Higher temperatures, longer exposure times, and increased fatigue loading considerably shortened the fatigue life of GFRP-RC beams, but CFRP-RC beams were barely affected at 400 °C. Both GFRP- and CFRP-RC beams lost almost all bearing capacity at 600 °C, aligning with the Tg results. Davalos et al. [168] exposed specimens to up to 30 freeze and thaw cycles at 60 °C, reinforced with GFRP and CFRP bars. The findings presented an approximately 18% reduction in the bond strength after exposure. Additionally, beams with CFRP and prestressed steel rebars were subjected to fatigue load at −30 °C. The key observations revealed slippage between concrete and rebars at loads with up to 90% of the ultimate strength capacity of the concrete beams at lower temperatures. It was also noted that concrete cubes strengthened with FRP under various temperatures increased microcracking in concrete specimens, which led to the decrement of the ultimate tensile load up to 15% [21,169]. This was attributed to the weakness of the FRP matrix under high temperatures, resulting in the bond failure. In addition, another experimental outcome showed a decrease in the bond strength of FRP bars of up to 40% at 100 °C, 70% at 140 °C, and 90% over 200 °C [156]. It was noted that glass fibers possess thermal insulation characteristics that are ten times less than those of basalt fibers. Temperatures below 0 °C may lead to modifications in the mechanical characteristics, causing microcracking in FRP material. Microcracks at low temperatures boost increased water retention at high temperatures, while lateral microcracks contribute to the expansion of frozen water within the cracks and gaps [78,153,158,161,164,170]. Such freezing exposures resulted in the material degradation by increasing brittleness and reducing debonding. All FRP composite materials lose their mechanical properties at elevated temperatures [95,153,161,163,167].

### 5.2. Fire Resistance

**Figure 5:**
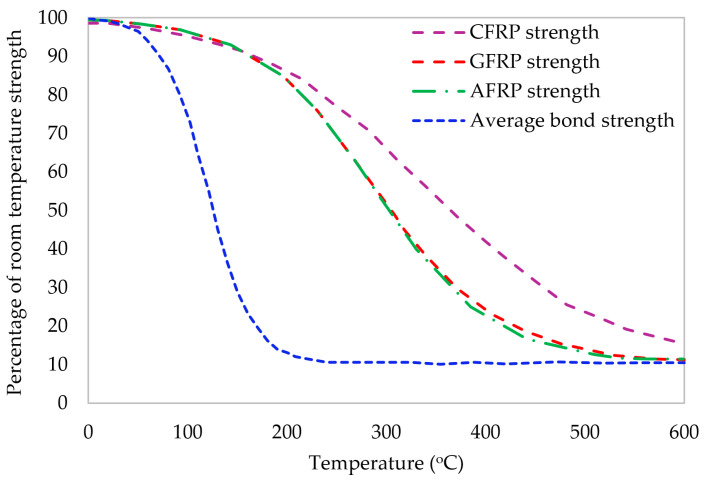
Temperature-dependent strength of CFRPs, GFRPs, and AFRPs.

Fire resistance poses a significant challenge, limiting the worldwide applications of FRPs. Addressing the response to fire exposure is becoming a crucial concern, especially considering the wide use of FRP in the fortification of RCC structures, especially in buildings located in fire-prone areas. The temperature-related strength of carbon, aramid, and glass fibers is shown in Figure 5 based on data analyzed from earlier research [171]. Enhancements in the FRPs’ fire resistance can be achieved through the utilization of fire-resistant polymers [27,28]. Despite these efforts, FRP materials must still indicate reliable behavior at high temperatures, aligning with fire design specifications to effectively safeguard buildings in the event of a fire [29]. Notably, glass fiber bars with thermoset resins containing halogen or bromine molecules are often used in coatings that resist corrosion and display a low rate of flame spread when exposed to external fire sources [31,172]. FRP thermal properties do not impact sectional temperature rises in FRP-fortified RC members. Neglecting temperature-induced bond degradation leads to unrealistic fire resistance estimates [21,156,161,162]. nonlinear bond–slip relations of Dai et al. [173] offer a more accurate assessment of the fire resistance. Considering the temperature-dependent variation of fire insulation properties is crucial for accurate assessments. Protchenko [174] examined FRP-reinforced beams under fire and concluded that BFRP-RC samples could not withstand two hours at elevated temperatures. Hybrid FRP-RC (HFRP-RC) beams gave an approximately 70% reduction in the strength, emphasizing the role of heating time. The failure mode differed between beams exposed to high temperatures, illustrating a significant effect of temperature on the bar strength. Additionally, BFRP-RC beam deflections were nearly twice as large as those in HFRP-RC beams, attributed to the “prestressing” effect of carbon fibers in HFRP bars during heating and cooling. In a different study, fiber-reinforced inorganic polymer (FRiP) was created with phosphate cement slurry instead of epoxy to increase the fire resistance in FRP composites. Based on the study, at room temperature, FRiP-to-concrete interfaces exhibited perfect bond failure, which is similar to that of conventional FRP systems. FRiP composites maintained more than 47% of their room temperature bond strength even after being exposed to fire. In comparison to its epoxy-based FRP equivalent, the phosphate-based FRiP efficiently strengthened concrete beams, retaining roughly 47% of its efficiency after the fire exposure [175]. In another investigation, the residual strength capacity of beams with only FRP reinforcement that were subjected to high temperatures was assessed. It included different types of reinforcements in the tensile zone. The findings demonstrated lower deflections compared to steel-reinforced beams. The tensile zone reached its ultimate strength capacity in all the samples exposed to high temperatures, resulting in failure. For BFRP bars, this strength capacity loss was roughly 43%, while for HFRP and nano HFRP bars, it was 40%. Despite the considerable reduction, beams reinforced with HFRP bars showed the strongest post-fire strength capacity, whereas the behavior of FRP-RC beams both during and after exposure to extreme temperatures was different [176]. The robust fire resistance observed in GFRP-strengthened RC beams can be attributed to the substantial concrete cover of 70 mm [31]. This aligns with findings from various literature reviews and theoretical studies [177,178,179,180,181]. The critical comparison and rankings between steel and FRP, especially their practicality and durability characteristics, are summarized in Table 9.

## 6. Strengthening Techniques

**Figure 6:**
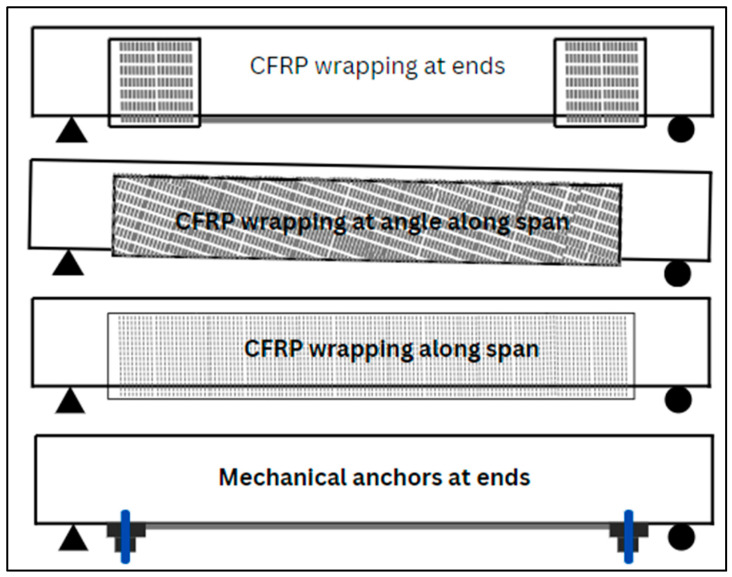
Structural strengthening using CFRP wraps and mechanical anchors.

**Figure 7:**
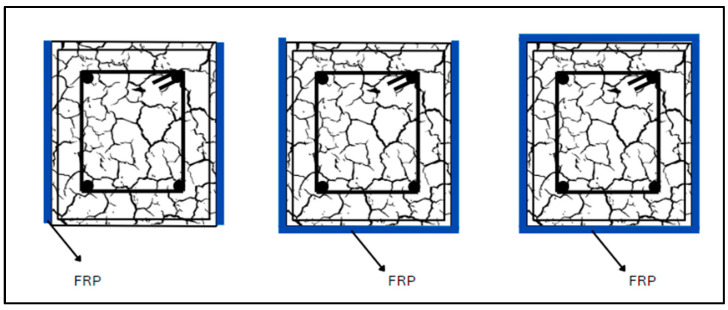
Strengthening with various wrapping schemes.

Various approaches have been used in the past to strengthen RC elements using FRPs. A variety of CFRP wrapping techniques and the application of mechanical anchors to strengthen the structures are depicted in Figure 6. A few new methodologies have been introduced, but they still require further investigation for a proper understanding of their effectiveness. Techniques like NSM and EBR are the most popular. Other techniques like external prestressing (EP), EBROG, and SC are also used for strengthening purposes. Three distinct techniques for wrapping FRP around structural elements are illustrated in Figure 7.

### 6.1. NSM

**Figure 8:**
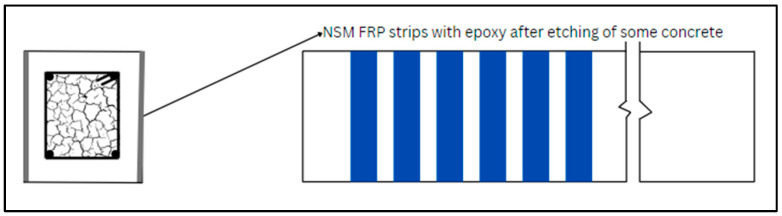
Shear and torsional strengthening with NSM technique.

**Figure 9:**
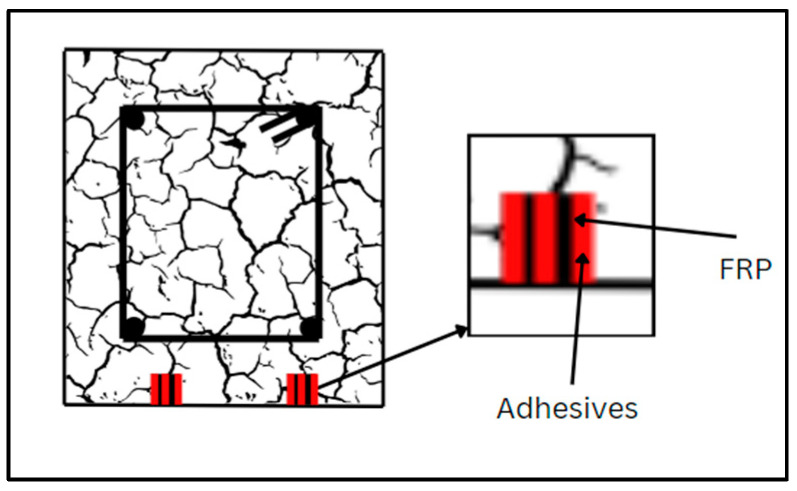
Grooves for NSM FRP flexural strengthening.

**Figure 10:**
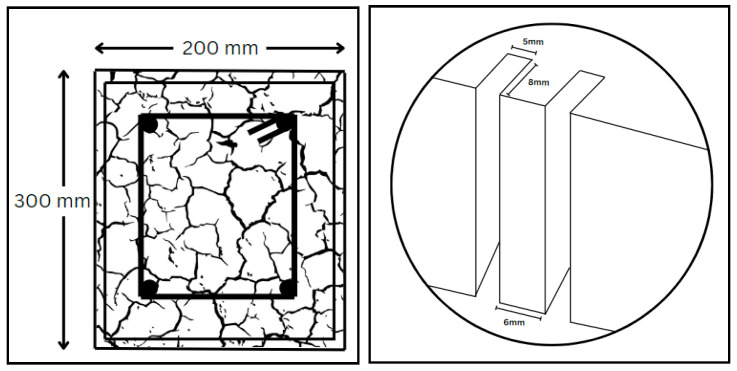
Cross-section size and groove dimensions for NSM FRP.

**Figure 11:**
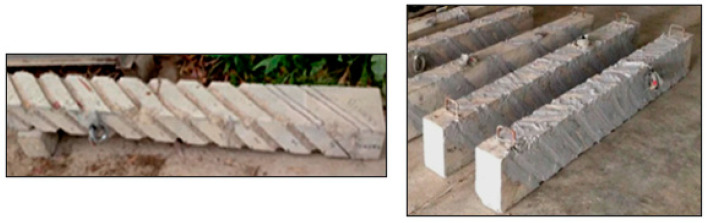
Spiral grooves on beams for NSM FRP [194].

The NSM approach is executed in several steps. First, using a diamond knife cutter splits 4–5 mm wide and 10–15 mm deep are meticulously cut on the concrete surfaces of the structural members, then the splits are cleaned utilizing compressed air, and the CFRP layers are further cleaned. Subsequently, epoxy resin is prepared as per the recommendations of suppliers. The cuts are then filled with epoxy resin, which is also applied to the laminate surfaces. Finally, the laminates are carefully inserted into the cuts, and any excess epoxy is removed, completing the NSM reinforcement process. Figure 8 displays the shear and torsional strengthening using the NSM technique. Several studies are available in which structural members are strengthened by FRP using the NSM technique [183,184,185]. Grooves filled with epoxy and FRP are indicated in Figure 9. Similarly, Figure 10 depicts the cross-section and groove dimensions of the structural elements. Palmieri et al. [30] investigated the fire performance of concrete beams, 3.1 m in span, strengthened/repaired with NSM FRP strips or bars (GFRP or CFRP). With bonding using epoxy (Tg between 62–65 °C), high Tg (82 °C) epoxy, or expansive cementitious mortar, the beams were insulated with U-shaped systems of five materials (20 to 100 mm thickness). Depending on the type of FRP, the beams may withstand loads up to 37% to 55% of their peak load at room temperature before failing the 2-h ISO 834 fire test. In the mechanical response, none of the strengthening systems exhibited evident separation or debonding, even though they had initially outperformed the adhesive Tg. Barris et al. [186] utilized the NSM technique to evaluate the flexural performance of GFRP-RC beams that were strengthened with CFRP strips and internally reinforced with GFRP bars. The outcomes demonstrated how well NSM CFRP performs to increase the flexural strength of beams reinforced with GFRP. Additionally, an analytical study examines the impact of various factors on the flexural capacity of NSM CFRP-strengthened RC beams, considering internal reinforcement with either steel or GFRP bars. Generally, a high reinforcement ratio and mechanical properties lead to improved flexural capacity, but parameter changes may result in different failure modes. Spiral grooves for FRP on concrete beams are depicted in Figure 11. Wang et al. [187] introduced two anchorage systems, additional ribs (ARs) and wire mesh mortar protection layer (WML), to resist premature detachment failures in the NSM FRP system. It was confirmed that these systems were able to improve the bond behavior of NSM FRP bars by testing twenty direct pull-out specimens. The bond strength was enhanced by the AR anchorage system and the WML anchorage system individually by up to 40.7% and 69.7%, respectively. The most successful combination of the two anchorage technologies produced the largest bond improvement of 114.3%. Zhang et al. [188] assessed the performance of T-beams strengthened with FRP bars under monotonic flexural loading, comparing the experimental findings with predictions from a first principles-derived flexural model. Multiple specimens were tested, evaluating the failure mode, cracking resistance, yielding, ultimate capacity, flexural stiffness, and ductility. Results illustrated a general increment in the flexural stiffness for strengthened specimens, particularly in the post-yield loading stage. Barris et al. [189] performed experiments investigating the effect of various NSM reinforcement layouts on the midspan deflection, crack spacing, and crack width in NSM FRP-RC beams. A few NSM FRP-RC beams, with one reference RC beam, were subjected to four-point bending until failure, using CFRP and GFRP rods. The findings revealed that NSM FRP reinforcement effectively reduces the deflection and crack width. Strengthened specimens exhibit larger crack formation phases, and crack width decreases with higher NSM FRP reinforcement ratios. Furthermore, cracks at the beams’ bottom surface are 10–25% wider than those at the height of the internal steel reinforcement. Table 10 provides an overview of a few relevant publications.

**Table 10:** Findings of selected studies on strengthening using NSM FRP method.

Author	Specimen	FRP	Ref.	FRP Type	Main Finding
Palmieri et al.	12 samples	CFRP/GFRP	[30]	Strips/ rebars	Under full-service load, 12 beams that were insulated with different systems and reinforced with NSM FRP bars were exposed to fire. The results showed that well-insulated components maintained some of their initial flexural capacity after fire exposure and that insulated NSM FRP strengthened beams can withstand fire for at least two hours.
Wang et al.	20 samples	BFRP/ CFRP	[187]	Bars	Two anchorage systems, ARs and WML, were used in this study to stop premature debonding during NSM FRP strengthening. Using ARs increased the bond strength by 40.7%, WML by 69.7%, and the combined application of both by 114.3%, according to a pullout test on 20 samples.
Zhang et al.	13 samples	CFRP/GFRP	[188]	Bars	Three control samples and ten RC T-beam samples with NSM FRP bars were examined. Failure mechanism, fracture resistance, yielding, ultimate capacity, flexural stiffness, and ductility were all studied. The flexural stiffness of strengthened samples often increases, according to the results, especially after the yielding stage. The results of experiments are compared to analytical predictions of the flexural strength, and a parameter for the flexural stiffness prediction model is proposed to account for decreases in the FRPs’ effective area when calculating the strength of the section.
Hassan and Rizkalla	9 samples	CFRP	[190]	Bars/sheet	Half-scale samples of pre-stressed concrete beams fortified with CFRP were tested. The tests revealed a 50% increase in both the stiffness and strength of the beams. The strength increment was affected by the type and layout of CFRP sheets/bars. Moreover, the study compared the economic aspect of several FRP systems, indicating that the overall cost of using CFRP sheets was approximately 25% cheaper than utilizing NSM rebars.
Ceroni et al.	21 samples	CFRP	[22]	Bars/ plates	Flexural tests were conducted on multiple concrete beams strengthened with both NSM bars and CFRP plates. The results demonstrated the effectiveness of FRP when externally bonded as sheets and/or used as NSM rebars. In beams fortified with CFRP sheets, the increment in failure load ranged from 18% to 50%, depending on the steel reinforcement ratio, but this increment was accompanied by a significant decrement in the ductility. Conversely, using an NSM system improved both the ultimate load capacity and ductility (by 45–70%) and suggested that CFRP sheets strengthened beams could achieve sufficient ductility by employing U-sheets.
El-Gamal et al.	10 samples	CFRP/GFRP	[191]	Hybrid (plates/bars)	Ten RC beams were cast and strengthened in flexure using various FRPs. All the beams exhibited an increase in the flexural capacity ranging from 30% to 133%. CFRP-strengthened beams attained the highest ultimate loads compared to those strengthened with GFRP sheets, while GFRP-strengthened beams displayed more ductile behavior. The test results obtained were according to predictions from ACI 440 provisions.
Al Bayati et al.	6 samples	CFRP	[192]	Bars	To evaluate the increase in the torsional strength achieved by various NSM FRP configurations and epoxies, six concrete members underwent testing. Based on the results, the ultimate torsional capacity of the beams increased by roughly 22–31% when the epoxy-based bonding material was utilized, and by 13–16% when the cement-based bonding agent was used. Furthermore, the study showed that while the torsional improvement of fully wrapped (closed) beams was greater than that of open (U)-wrapped concrete beams, in certain situations the adoption of a U-shaped strengthening plan might be more advantageous.
Yu and Kodur	4 samples	CFRP	[193]	Bars	Four concrete T-beams, strengthened with NSM FRP, were subjected to fire exposure and service loading conditions. The results of the fire tests illustrated that NSM CFRP-strengthened beams can achieve a fire resistance of 3 h under fire exposure, even without any fire insulation.

### 6.2. EBR and EBROG

**Figure 12:**
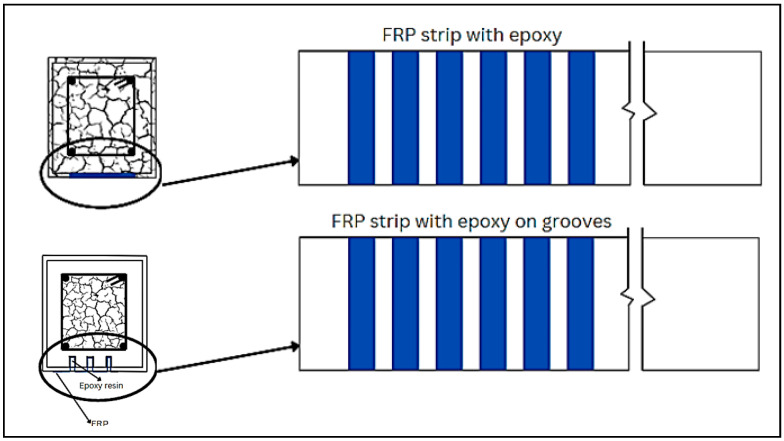
EBR and EBROG with FRP strips for shear strengthening.

**Figure 13:**
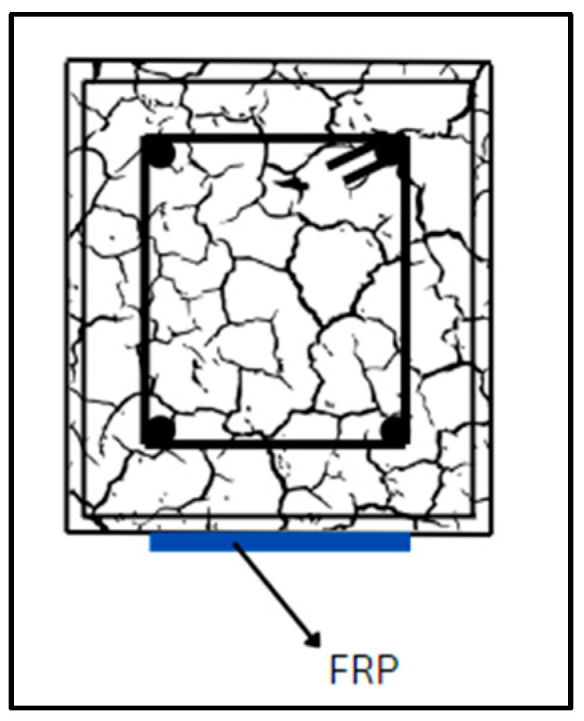
EBR-FRP for flexural strengthening.

**Figure 14:**
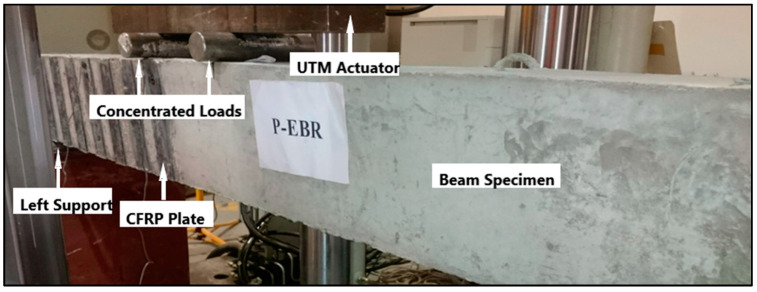
Grooving and CFRP sheets on beams [209].

Other very useful methodologies for strengthening of RC members are EBR and EBROG. Figure 12 depicts EBR and EBROG techniques for shear strengthening of the concrete beam. The process of EBR strengthening is done in a few steps. First, the surfaces of the beam where the laminates would be bonded are prepared, emery paper is employed to remove the external paste, and any residue is removed using compressed air. A coat of primer is then applied to even out the concrete surface and enhance the strength capacity of the substrates. Finally, the laminate sheets are attached to the beam surfaces utilizing epoxy resin, as indicated in Figure 13. In EBROG, a groove or recess is made on the surface of concrete for the strips or rods to be bonded. A beam strengthened with CFRP using the EBROG method is displayed in Figure 14. Multiple studies have examined the effect of strengthening of RC members with this technique [195,196,197,198,199,200,201,202]. Wu et al. [203] carried out double lap-shear tests on concrete blocks strengthened with CFRP sheets bonded externally using an epoxy adhesive with a relatively low Tg (ranging from 34 °C to 38 °C). As the temperature increased beyond Tg, a steady decline in failure load was noted, with only 40% retention at 50 °C. Similarly, Gamage et al. [204] performed single lap-shear tests between concrete and CFRP sheets bonded with epoxy resin showcasing substantial strength reductions with increasing temperature, particularly in the 50 to 75 °C range. Beyond this, the normalized bond strength decreased to less than 25% of the outside temperature. This reduction was accompanied by a shift in failure modes, from a combination of bond failure and concrete rupture (up to 50 °C) to peeling-off adhesive failure (above 60 °C). For the utilization of prestressed CFRP composites in reinforcing concrete structures, the novel EBROG technique was explored and compared to the traditional EBR method. The outcomes demonstrated that the EBROG approach significantly improved the bond resistance, with 2.4 times increase over EBR. With this technique, the bulk concrete saw considerable crack formation, which improved the prestressed joints’ fracture energy and bond resistance. Sabzi et al. [38] investigated eight RC beams with different reinforcement arrangements and classified them into the EBR and EBROG methods; the beams exhibited different failure modes based on strengthening techniques and concrete strength. NSC beams with EBR faced FRP debonding, while EBROG resulted in concrete cover separation. In HSC beams, both methods led to FRP debonding. EBROG considerably increased the ultimate load, mid-span deflection, and load-deflection curve area compared to EBR in HSC beams. Torabian et al. [205] explored the efficacy of the EBROG method in flat slabs under realistic conditions. Two layouts, representing roof-level and intermediate floors, were tested with concentric monotonic loading. The strengthening solutions involved CFRP sheets and two FRP bonding techniques, EBR and EBROG. The results revealed the EBROG’s effectiveness in delaying debonding for both layouts, with the load capacity increasing by 36% (cross layout) and 15% (grid layout) compared to EBR. In another study, the impact of freeze–thaw cycles on the bond strength between CFRP strips and concrete was studied. A total of 18 specimens were prepared, with 12 strengthened using CFRP strips subjected to 200 and 500 freeze–thaw cycles. Strengthening methods included EBR and EBROG. Results illustrated the improved bond performance in EBROG-strengthened specimens compared to EBR. EBR specimens showed 3% and 9% bond strength decreases, respectively, while EBROG specimens experienced increments ranging from 7% to 19%, respectively [206]. Moghaddas and Mostofinejad [201] reported a novel FRP concrete bond strength model through nonlinear regression on experimental data, verified using statistical tools and variance analysis. A total of 154 single lap-shear tests on 136 EBROG samples and 18 EBR specimens were conducted and optimized utilizing response surface methodology and I-optimality criteria. The study compared EBROG and EBR specimens, revealing debonding failure in both. However, EBROG indicated superiority, achieving a 31% average bond strength improvement and delayed debonding. The proposed EBROG bond strength model adapted the Chen and Teng model, initially used for EBR. Table 11 provides an overview of some relevant publications.

**Table 11:** Main findings of relevant studies on strengthening using EBR and EBROG.

Author	Sample	FRP	Ref.	FRP Type	Main Finding
Gamage et al.	10	CFRP	[204]	Sheets	This study evaluated issues related to the bond performance at elevated temperatures using a shear test method. The experimental program measured the bond strength at increasing epoxy temperatures, revealing a rapid loss of the strength beyond 60 °C.
Sabzi et al.	8	CFRP	[38]	Sheets/bars	This investigation assessed the influence of reinforcement arrangement and concrete strength. The findings demonstrated that conventional concrete beams strengthened with the EBR and EBROG methods exhibit FRP debonding and concrete spalling failure modes, respectively. Conversely, in HSC beams, both the EBR and EBROG methods resulted in FRP debonding failure mode. Moreover, HSC specimens treated with the EBROG method displayed a noticeable increase in the ultimate load-carrying capacity and mid-span deflection compared to the EBR method.
Torabian et al.	5	CFRP	[205]	Sheets	The EBROG technique effectively delays debonding. Compared to EBR, EBROG increases the load capacity by 35% when FRP sheets are attached to the joint and by 15% when bonded away from the joint. Debonding strains are substantially higher in EBROG.
Moghaddas and Mostofinejad	154	GFRP	[201]	Sheets	Debonding failure is observed in all specimens, illustrating the excellence of EBROG over EBR. An average enhancement of 30% in the bond strength and postponed debonding is achieved with the EBROG method.
Mostofinejad and Kashani	32	CFRP	[207]	Sheets	The results revealed that while surface preparation does not entirely prevent debonding, it can delay it, leading to an increase in beam-carrying loads of up to 12%. Additionally, the grooving method outperforms surface preparation, as none of the beam specimens strengthened using this method experienced FRP strip detachment. This resulted in the disappearance of shear weaknesses, making the flexural failure the dominant mode.
Mostofinejad et al.	20	CFRP	[208]	Sheets	The effectiveness of the EBR, EBROG, and EBRIG methods in flexural strengthening of RC beams was comparable with a single layer of FRP sheet. However, with two FRP layers, beams strengthened utilizing the grooving method in the form of both the EBROG and EBRIG techniques exhibited up to 25% higher peak loads compared to the EBR method. In addition, with three FRP layers, the increase was 20% and 25% for the EBROG and EBRIG strengthening techniques, respectively, compared to the EBR method.

### 6.3. EP

**Figure 15:**
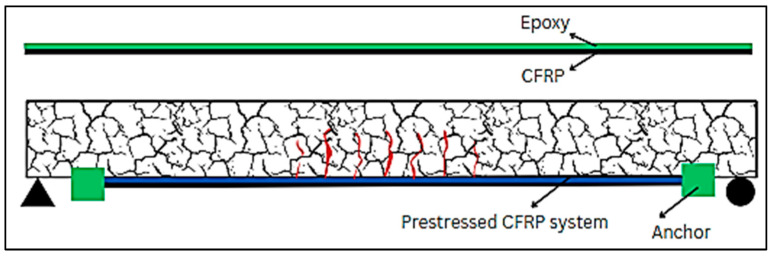
Strengthening with externally prestressed CFRP and mechanical anchors.

**Figure 16:**
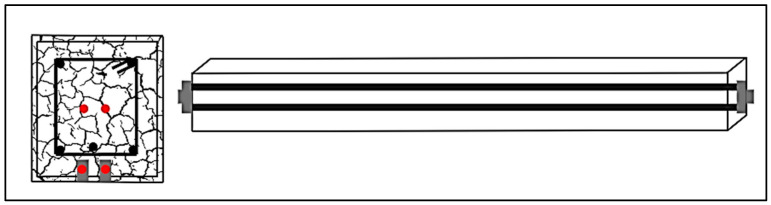
Strengthening with externally prestressed FRP.

**Figure 17:**
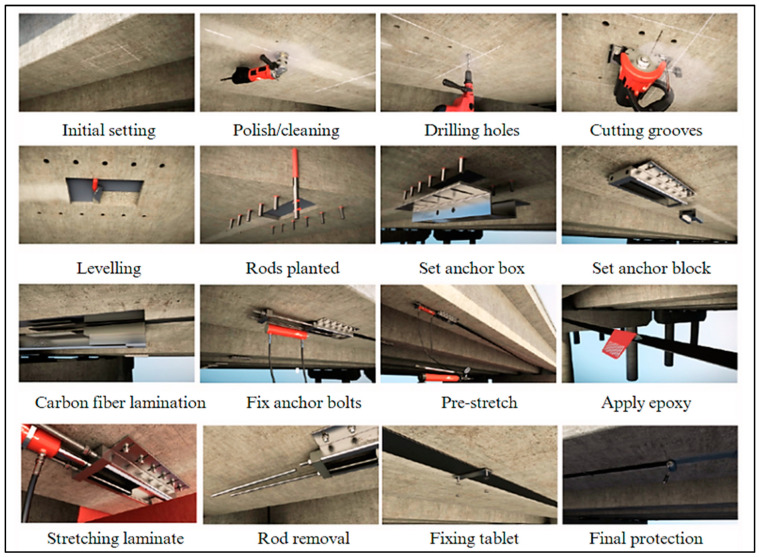
Steps used by Horse FRP for strengthening with externally prestressed CFRP laminates [212].

EP is a novel and effective method of strengthening of existing structures, such as concrete or steel columns and beams. The strengthening of structural elements, using externally prestressed CFRP and mechanical anchors, is demonstrated in Figure 15. Similarly, strengthening with only externally prestressed FRP is shown in Figure 16. To eliminate the issues associated with corrosion, FRPs are used instead of steel in external tendons, though anchoring the external FRP tendons is still a big problem. Several studies are available in which this technique (EP) has been used [210]. Gao et al. [211] tested six slab samples, including one reference slab without EP, to evaluate the feasibility and effectiveness of the new structural strengthening method. The remaining five slabs were strengthened with external post-tensioned GFRP tendons utilizing the new anchoring system, varying in prestressing level and tendon ratio. Measurements were taken at the first cracking moment, at the bar yielding moment, and at the ultimate moment, resulting in improved slab stiffness during loading. The results indicated that strengthening with external tendons resulted in maximum increases of 121% and 103% for the first cracking and ultimate moments, respectively. Moreover, this approach decreases tensile stresses in concrete and bonded reinforcement, leading to thinner and denser cracks and the improvement of the ultimate strain in the top concrete fiber. Increased prestressing force and the number of external tendons further amplify these positive effects. Figure 17 displays the step-by-step procedure for applying externally prestressed CFRP for structural strengthening [212]. Kim et al. [213] carried out research to address prestress losses and aging in prestressed concrete structures (PSCs); strengthening methods such as NSM and EP were used, utilizing FRPs, particularly CFRPs, for their non-corrosive and high tensile strength properties. The NSM and EP methods remarkably improved the stiffness by 50–60% compared to the control PSC specimen. Notably, the EP method, especially when combined with prestressing, revealed the most effective strengthening outcome. In another research project, they investigated four strengthening methods, NSM, EP, EBR, and SE, using FRP due to its high tensile strength and non-corrosive nature. These methods employed some strengthening materials like CFRP, GFRP, and prestressing strands. The concrete EP and NSM(P) methods exhibited almost double the stiffness of the non-strengthened specimen. Due to the brittleness of the EP and EBR methods, the NSM(P) method, with ductile behavior, is considered the most effective. Lou and Karavasilis [214] assessed the substitution of steel tendons with external FRP tendons in prestressed steel–concrete composite (PSCC) girders. The variables included tendons (CFRP, AFRP, and steel) and prestress level (0 to 60%). The results demonstrated similar behavior between CFRP and steel, while AFRP tendons showed lower ultimate load and higher deformation capacity. Prestress level minimally influences the moment at the center of continuous PSCC girders due to secondary moments. Although the EP method has proved to be beneficial for strengthening of the existing structural members, there is still a huge research gap to be filled. The findings of a few relevant publications are presented in Table 12.

### 6.4. SC

**Figure 18:**
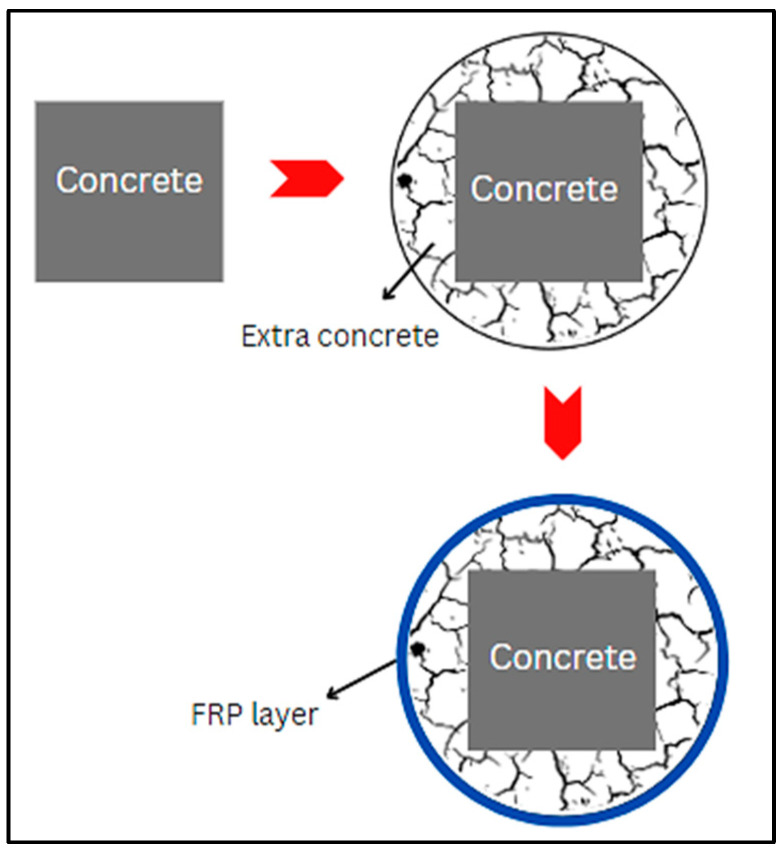
Section modification of existing section with concrete and FRP layer.

Another approach for strengthening of the structural members is SC. In this method, the corners of the members are converted into round surfaces, as shown in Figure 18. Several studies have demonstrated the efficacy of FRP jacketing for strengthening of circular RC columns but exhibited less effectiveness for rectangular or square columns because of the flat sides and sharp edges [216,217]. To address this limitation, the SC method has been proposed, involving modifying flat sides into moderately curved sides before FRP application. Zeng et al. [217] studied the SC method using ultra-high-performance engineered cementitious composites (UHP-ECC) and confinement of FRP. Eleven FRP-constrained square RC columns with SC were investigated utilizing UHP-ECC to evaluate parameters like additive concrete strength *a/d* ratio and FRP thickness. The findings revealed that the SC approach, when combined with FRP jacketing and UHP-ECC, enhances the load-bearing capacity by 52% and the section’s increment by 17%. An assessment of the current design-oriented stress–strain models highlights the need for more research to improve the models for constrained concrete in curvilinearized square columns (CSCs) and raises the possibility that the ultimate strength may have been underestimated. Zhu et al. [218] presented systematic experimental findings on CSCs and curvilinearized rectangular columns (CRCs). Testing 16 smaller and 10 larger FRP-enclosed square concrete columns, with or without SC, under axial compressive loading, the study reported a limited size effect on FRP-confined CSCs. The precision of present stress–strain models for FRP-enclosed concrete in CRCs was assessed, highlighting the need for an improved stress–strain model for design purposes. In another research, axial compression tests on large FRP-confined CRCs were performed. The tests examined key variables such as *a/d* of the curved surfaces, the corner radius ratio, and the aspect ratio of the section. The results displayed that the slope of the linear second portion of the stress–strain curve for FRP-enclosed concrete in a CRC is significantly larger than that of an associated column without SC, illustrating the effectiveness of the SC method [219]. Aules et al. [37] studied the impact of shape alteration on the efficacy of CFRP strengthening in square-section concrete members. Examined parameters included the shape modification type (edge rounding, circularizing, and curvilinearizing square sections), strengthening layouts (fully (closed) and partial), and the number of CFRP layers. The results indicated that the circularized shape showed the highest increment in the compressive strength, and the complete confinement strengthening layout was more efficient than partial confinement. Doubling the number of CFRP layers proportionally increased the compressive strength. Modifications to a circular and curvilinear section greatly improved the energy absorption and deformation capacity compared to edge-rounded confined specimens. The failure mode of strengthened samples remained consistent across different shape modifications. A few selected publications on section modifications are tabulated in Table 13.

## 7. Future Research Directions

Unlike the NSM or EBR techniques, there is a huge research gap for new approaches such as SC and EP. From previous experimental and analytical studies, it is evident that future research should be focused on the long-term behaviors of various FRP reinforcements and strengthening techniques, constitutive modeling, and numerical studies. From this review, a few future research directions are summarized as follows:

**Numerical/Analytical Study**

- As discussed earlier, there is a huge gap in the research on the enhancement of stress–strain models for FRP-confined concrete such as confined concrete in CSC. The existing design-oriented stress–strain models available can underestimate the ultimate strength of confined concrete in CSCs. Furthermore, predictive machine learning models can be trained to make the work easier.
- Detailed numerical analysis is required for the validation of all the structural strengthening techniques discussed above for better applicability and acceptance of the methods.

**Long-Term Behavior of FRP**

- Although many studies that evaluate the short-term performance of FRP-strengthened concrete under different conditions are available, the long-term behavior and performance of FRP-strengthened concrete are still to be studied. Advanced structural health monitoring techniques (sensing devices) should be applied to the FRP-strengthened members to assess their long-term behavior.

**Environmental Aspects**

- Various studies [87,88,98] have explored the durability of FRPs, yet the combined impact of particularly harsh environmental conditions in wastewater treatment plants, chemical plants, and nuclear plants has not been thoroughly investigated. These conditions can be direct exposure to UV rays, temperature fluctuations, freeze–thaw cycles, and exposure to acidic–alkaline environments. Therefore, there is a need to assess the durability of FRP-strengthened RC structures under the simultaneous influence of these challenging conditions.
- Additional research into the life cycle assessment of different strengthening techniques using various FRPs is necessary for evaluating environmental performance and enhancing future strengthening methods.
- To keep the environment clean, research on green alternatives to traditional polymer matrices like epoxy and polyester is crucial. These alternatives can remarkably reduce the environmental effects related to significant energy inputs in the manufacturing process and the potential toxicity of conventional polymers.

**Structural Testing and Design**

- There are several research limitations and challenges in every strengthening technique when subjected to combined loadings such as fatigue, cyclic loading, seismic events, and impacts. Conducting research that considers these coupled loading conditions would be valuable for the practical application of FRP strengthening techniques in real-world scenarios.
- A very limited number of studies with other types of FRPs such as BFRP and AFRP are available. A comparative study of structural strengthening methods utilizing various types of FRP material can result in a more economical, sustainable, and safer structure. Additionally, there is a research gap in the torsional behavior of FRP-strengthened members which must be filled to properly examine their performance under different conditions.
- For some new strengthening methods, the accurate design procedures have not been clearly defined. Some common challenges are anchorages, member and material interface differences, step differences between the member and anchorage, and variations in material properties among different manufacturers. Future research must collect and use more precise data from design and building sites to address these concerns.

## 8. Conclusions

The above-discussed literature review demonstrated recent advancements in the strengthening techniques of existing RC structures and members. It also revealed the important role of FRPs in structural rehabilitation and strengthening. With some extraordinary properties such as their non-corrosive, lightweight nature, and high strength, FRPs are becoming popular in the construction industry, particularly as a replacement for structural strengthening materials. Since their initial usage in the 1960s, many new design methodologies and structural strengthening techniques have been introduced worldwide. The researchers performed different types of analysis of structural members strengthened with FRPs under different loading and environmental conditions.

Different types of FRPs used in the construction industry have various purposes. For instance, the mechanical properties of CFRP are better compared to all other typologies. Also, CFRP performs better in the worst environmental conditions. One of the major drawbacks of CFRP is high carbon emissions. AFRP performs better in marine environments compared to other types. Moreover, for structural strengthening, CFRP with high tensile strength and elastic modulus is an effective option, among others. Apart from synthetic FRPs, natural FRPs are also utilized in the construction industry. Although natural FRPs do not have the same properties as synthetic FRPs, using a mixture of both synthetic and natural FRPs is a better alternative. According to a few studies, one of the defining factors in the mechanical properties of synthetic FRPs is fiber orientation. For example, the unidirectional alignment of FRP fibers enhances the flexural and tensile strengths.

Studies have reported that a combination of different FRPs can improve the performance of structures. For instance, a combination of CFRP and GFRP could enhance the flexural capacity of structural members by more than 100%.

Research works have illustrated that FRPs exhibit high tensile strength, elastic modulus, and fatigue resistance. They also display low abrasion and low coefficient of thermal expansion. In addition, the shear and flexural capacities of structural elements are enhanced by 15–20% and 20–80%, respectively. Different configurations of FRPs such as U-shaped jackets can improve these capacities further. Of the various structural strengthening techniques, NSM and EBR are the most popular among constructors. These techniques can alter the failure modes of structural elements. As an example, they could alter the brittle shear failure to the bending shear failure of the structural element. Although, in terms of failure, NSM is considered the most effective and safest structural strengthening technique, EBR is popular owing to its ease of installation.

## Figures and Tables

**Table 1:** Characteristics of GFRP.

Trade	Tensile Strength (MPa)	Young’s Modulus (GPa)	Ultimate Tensile Strain
Aslan	690	40.8	0.017
Nefmac	600	30	0.020

**Table 2:** Characteristics of CFRP.

Trade	Tensile Strength (MPa)	Young’s Modulus (GPa)	Ultimate Tensile Strain
Aslan	2068	124	0.017
Nefmac	1200	100	0.012

**Table 3:** Characteristics of AFRP.

Trade	Tensile Strength (MPa)	Young’s Modulus (GPa)	Density (g/cm^3^)
Kevlar	2.3–3.4	70–43	1.44–1.47
Heracron	2.8	123	1.44

**Table 4:** Characteristics of BFRP.

Trade	Tensile Strength (MPa)	Young’s Modulus (GPa)	Elongation (%)
Rockbar	1000	50	2.24
BCR	1100	70	2.2

**Table 5:** Physical characteristics of FRP composites.

Material	Strength of Fiber (MPa)	Strength of Laminate (MPa)	Density of Laminate (g/cc)	Strength to Weight Ratio	Young’s Modulus (GPa)
GFRP	3450	1500	2.3–2.5	565	30–40
CFRP	4128	1600	1.8–2.1	1013	125–180
AFRP	2757	1430	1.44	993	70.5–112.4
BFRP	3792	1100	2.6–2.8	1000	70–90

**Table 6:** Mechanical properties of various FRPs.

Reinforcement	Yield Strength (MPa)	Density (g/cm^3^)	Tensile Strength (MPa)	Specific Gravity	Elastic Modulus (GPa)	Strain at Break (%)
Steel	500–500	7.8–8.1	-	7.8	200	-
GFRP	600–1400	2.12–2.75	480–1600	1.5–2.5	35–51	1.2–3.1
BFRP	1000–1600	2.15–2.70	1035–1650	2.7–2.89	45–59	1.6–3.0
AFRP	1700–2500	1.28–2.6 1.39–1.45	1720–2540	1.38–1.39	41–125	1.9–4.4
CFRP	1755–3600	1.55–1.76	1720–3690	1.0–1.1	120–580	0.5–1.9

**Table 7:** Selected studies on RC strengthened by FRP in shear.

Author	Ref.	Sample	FRP Type	Cross Section (mm)	No. of Layers	Thickness of Layers (mm)	Midspan Deflection at Maximum Load (mm)	Failure Mode	Experimental Shear Strength (kN)
El-Sayed	[121]	C-1.0 (control)	-	150 × 300	-	-	2.8	Shear	46.5
		PL-1.0-0.43	Low modulus plates	150 × 300	1	1.4	2.48	Shear	59
		PL-1.0-0.85	Low modulus plates	150 × 300	2	1.4	2.57	Shear	61.5
		PL-1.0-0.36	Low modulus plates	150 × 300	1	1.4	2.27	Shear	58.5
		S-1.0-0.27	Carbon sheets	150 × 300	3	0.3	2.59	Shear	50
		C-1.5 (control)	-	150 × 300	-	-	2.17	Shear	44.5
		PL-1.5-0.85	Low modulus plates	150 × 300	2	1.4	1.94	Shear	60
Li et al.	[122]	AC (control)	-	150 × 300	-	-	3.13	Deep beam	253
		AS (control)	-	150 × 300	-	-	3.41	Deep beam	258
		A150	CFRP-MF	150 × 300	-	0.167	3.74	Deep beam	260
		A80	CFRP-MF	150 × 300	-	0.167	3.94	Deep beam	272
		A55	CFRP-MF	150 × 300	-	0.167	4.6	Deep beam	282
		BC (control)	-	150 × 300	-	-	3.94	SC	74
		BS (control)	-	150 × 300	-	-	4.63	SC	108
		B150	CFRP-MF	150 × 300	-	0.167	4.91	SC	112
		B80	CFRP-MF	150 × 300	-	0.167	5.23	SC	124
		B55	CFRP-MF	150 × 300	-	0.167	5.94	SC	129
		CC (control)	-	150 × 300	-	-	4.56	Flexural shear	58
		CS (control)	-	150 × 300	-	-	6.14	Flexural shear	94
		C150	CFRP-MF	150 × 300	-	0.167	6.27	Flexural shear	97
		C80	CFRP-MF	150 × 300	-	0.167	6.51	Flexural shear	109
		C55	CFRP-MF	150 × 300	-	0.167	7.51	Flexural shear	118
Faramarzi et al.	[123]	U/3 (control)	-	75–200 × 325	-	-	10	Diagonal tension	75.5
		S/3	CFRP bars	75–200 × 325	-	-	11.3	Diagonal tension	103.6
		U/1.9 (control)	-	75–200 × 325	-	-	6	SC	103.9
		S/1.9	CFRP bars	75–200 × 325	-	-	10	SC	134.7
Murad	[124]	SC (control)	-	150 × 200		-	18	Shear	88
		S0	CFRP sheets at 0°	150 × 200	N/A	0.166	16	Shear	93
		S90	CFRP sheets at 90°	150 × 200	N/A	0.166	20	Shear	100
		S45	CFRP sheets at 45°	150 × 200	N/A	0.166	20	Shear	103
		S60	CFRP sheets at 60°	150 × 200	N/A	0.166	25	Shear	91

**Table 9:** Comparison and rating of FRP versus steel [182].

**Properties**	**Benefit Rating**		**Scale**
	**FRP**	**Steel**	5 = Very high 4 = High 3 = Medium 2 = Low
Weight	5	2	
Strength/stiffness	4–5	4	
Corrosion resilience	4–5	3	
Ease of construction work	5	3–4	
Ease of repair	4–5	3–5	
Fire behavior	3–5	4	
Ease of mobility	5	3	
Toughness	4	4	
Maintenance	5	3	

**Table 12:** Summary of selected studies on strengthening using externally prestressed FRP.

Author	Sample	FRP	Ref.	FRP Type	Main Finding
Gao et al.	6	GFRP	[211]	Tendon	Slabs were tested until the point of failure and their flexural behaviors and failure modes were examined in detail. The tests indicated that the proposed anchoring methodology is reliable and efficient. In the end, a method for stress calculation in the tendons was also proposed.
Kim et al.	12	CFRP	[213]	Sheets	All NSM fabricated samples and those utilizing the EP method exhibited the stiffness increase of 50–60% compared to the control PSC specimen. The EP method, when combined with prestressing, displayed the most effective strengthening results.
Wang et al.	4	BFRP	[36]	Tendon	All strengthened RC beams indicated failure due to concrete crushing, featuring satisfactory crack patterns and superior ductility compared to the control beam. This underscores the concurrent action of internal steel reinforcements and external BFRP tendons during loading.
Tran et al.	32	CFRP/HM-CFRP, AFRP, BFRP, GFRP	[215]	Tendon	CFRP tendons with an elastic modulus (E) of 145 GPa were determined as the optimal option for replacing steel in PSCBs with external tendons. This is attributed to their ability to provide similar strength and ductility to steel tendons in PSCBs. On the other hand, the utilization of high-modulus CFRP tendons, for instance, E_p_ = 200 GPa, resulted in the enhanced stiffness and strength for PSCBs but came at the expense of significantly reduced ductility in the beams.

**Table 13:** Findings from relevant studies on section modification.

Author	Ref.	Main Finding
Zeng et al.	[217]	The stress–strain curve for FRP-enclosed concrete within a CRC had a substantially steeper linear second section than the other rectangular column. Furthermore, when the rise to span ratio (*r/s*) increased, so did the ultimate axial tension. When compared to a corresponding rectangular column, CRCs with the *r/s* ratios of 0.05, 0.066, and 0.1 showed improvements in the ultimate axial stresses of 20%, 40%, and 70%, respectively. The corner radius ratio of 0.2 and the *r/s* value of 0.066 seem to be the best choices for a discernible increase in the final axial stress and effectiveness of the FRP encasement.
Zhu et al.	[218]	The impact of size variation is restricted in these FRP-confined CSCs, and the compressive strength can be noticeably improved through the SC method, except for the ultimate axial strain which was not considerably affected.
Pan et al.	[220]	With the increment in the slenderness ratio, the effect of strengthening decreased. The load-carrying capacity of FRP-covered columns was 20% more than the ordinary reinforced RC column when the slenderness ratio was limited under 18.
Teng and Lam.	[221]	The efficacy of confinement decreased as the major to minor axis length ratio reduced, and the stress-strain curve descended when the effective confinement ratio was 0.11 or less. Also, a strength model was established for FRP-confined concrete in elliptical columns.
Zhao et al.	[222]	The highest and lowest increments in the compressive strength were 125% and 88% for the samples with the *r/s* ratios of 0.1 and 0.05, respectively.
Yan et al.	[223]	The FRP-confined CSC and CRC’s post-peak hardening behavior was caused by SC/section ellipticalization. When compared to FRP jacket-confined elements, SM square/rectangular columns with post-tensioned FRP shells demonstrated a greater axial compressive strength and energy absorption.
Parvin and Shroeder.	[224]	The efficacy of CFRP covering was significantly decreased when loaded eccentrically compared to when loaded concentrically, and CFRP covering was more efficacious in the axial direction than the hoop direction for columns loaded eccentrically.

## Data Availability

The data used for this study are available in the article.
